# A unified approach for determining the ultimate strength of RC members subjected to combined axial force, bending, shear and torsion

**DOI:** 10.1371/journal.pone.0175834

**Published:** 2017-04-17

**Authors:** Pu Wang, Zhen Huang

**Affiliations:** School of Naval Architecture, Ocean and Civil Engineering, Shanghai Jiaotong University, Shanghai, P.R. China; Beihang University, CHINA

## Abstract

This paper uses experimental investigation and theoretical derivation to study the unified failure mechanism and ultimate capacity model of reinforced concrete (RC) members under combined axial, bending, shear and torsion loading. Fifteen RC members are tested under different combinations of compressive axial force, bending, shear and torsion using experimental equipment designed by the authors. The failure mechanism and ultimate strength data for the four groups of tested RC members under different combined loading conditions are investigated and discussed in detail. The experimental research seeks to determine how the ultimate strength of RC members changes with changing combined loads. According to the experimental research, a unified theoretical model is established by determining the shape of the warped failure surface, assuming an appropriate stress distribution on the failure surface, and considering the equilibrium conditions. This unified failure model can be reasonably and systematically changed into well-known failure theories of concrete members under single or combined loading. The unified calculation model could be easily used in design applications with some assumptions and simplifications. Finally, the accuracy of this theoretical unified model is verified by comparisons with experimental results.

## Introduction

Failure of reinforced concrete (RC) members in extreme loading events is typically caused by different combinations of axial force, bending, shear and torsions, and the failure mechanisms can be highly complex. Compared to steel structural design and calculation theory, the design and calculation theory of RC members under the combined actions of tensile/compressive axial force, bending, shear and torsion is relatively imperfect and a unified failure theory that can be used worldwide has yet to be developed. The majority of design codes use experimental formulas to calculate the bearing capacity of RC members under combined loading actions; however, experimental data and theoretical formulation for these cases are lacking.

Currently, the theories for RC members subjected to axial forces and bending moments used in various countries are mostly identical, and this theory is widely accepted. However, there are many different theories and design methods for RC members subjected to shear and torsion loading. The existing research on the ultimate strength of RC members subjected to a combination of the four loads is far from conclusive, and experimental research is lacking, which makes theoretical research even more difficult.

Many theories have been established for RC members subjected to combined loads. These theories mainly include statistical analysis methods, truss models and limit equilibrium theory. Because the failure mechanisms of RC members are highly complex under combined loading, statistical analysis methods are widely used in construction applications. Statistical analysis methods have been used to establish semi-experimental equations based on regression analyses of experimental results. Statistical analysis methods typically aim to prevent the members from experiencing brittle shear failure but do not attempt to accurately predict the ultimate strength. Statistical analysis methods create concise equations and are convenient for many applications. However, statistical analysis methods lack mechanical models and require vast experimental data.

Truss models have led to numerous advancements in research on the shear and torsion loading of concrete over the past several decades. The most representative theories are compression field theory (CFT) and softened truss model theory. In 1973, Collins first proposed the deformation compatibility condition for RC members under shear, which is the Mohr deformation compatibility condition [1]. Mitchell and Collins [2] subsequently established CFT for RC members under shear and torsion in 1974 using the Mohr deformation compatibility condition, equilibrium condition and uniaxial stress-strain relationship of concrete.

In 1981, Vecchio and Collins [3] quantitatively analysed the softening effect in the stress-strain relationship of concrete in a multi-axial stress state and introduced the softening stress-strain relationship into CFT, which was a significant breakthrough in the research on RC members under shear. CFT assumes that the concrete tension stress is zero after cracking even though the residual tensile stress in the concrete between the inclined cracks is not actually zero. To consider the influence of the residual tensile stress, Vecchio and Collins [4] proposed the modified compression field theory (MCFT) based on CFT. The key improvement of MCFT is that it considers the tension stiffening of RC elements between the inclined cracks and restricts the concrete tensile stress of the concrete by checking the local equilibrium at the cracks. MCFT provides a more accurate evaluation of the ultimate strength than CFT. Vecchio experimentally determined that the directions of principle strains in concrete differed from those of the principle stresses when the deformation of concrete was extremely large. Therefore, Vecchio [5–7] introduced shear slip into the deformation compatibility condition to consider the difference between the stress and strain directions and also established the disturbed stress field model (DSFM).

In 1988, Hsu [8] established the rotating angle-softened truss model (RA-STM) based on the equilibrium condition, deformation compatibility condition and softened stress-strain relationship. The RA-STM is similar to MCFT; however, the RA-STM uses the experimental stress-strain relationship of the reinforcement embedded in the concrete instead of the relationship of the bare reinforcement and does not require checking the local equilibrium at the cracks. Hsu found that the RA-STM was only valid when the degrees of the inclined cracks were between 33° and 57° and that it could not consider the shear capacity of concrete. Pang and Hsu [9] established the fixed angle-softened truss model (FA-STM) in 1996. The FA-STM has a larger scope of application and can consider the shear capacity of concrete. The FA-STM is considerably more complex than the RA-STM because the equilibrium condition and deformation compatibility conditions are more complex; in addition, FA-STM adopts an additional shear constitutive relationship for cracking concrete. Hsu and his team [10] made significant developments in truss model theory and referred to their work as a unified theory of reinforced concrete.

The ultimate strength of RC members can also be investigated via limit equilibrium theory. In 1968, Gvozdev [11] established the limit equilibrium theory for warped failure surfaces considering the equilibrium and constitutive relations. Gvozdev established equations for the ultimate strength of RC members based on limit equilibrium theory by making assumptions about the shape of the failure surface. Huang [12, 13] established equations for the ultimate strength of RC members with box sections and rectangular sections by modifying the limit equilibrium theory for a warped failure surface. However, in traditional limit equilibrium theory, it is often difficult to determine the shapes of the failure surfaces, and the shear capacity of concrete is neglected; these simplifications lead to errors. Nielson [14] proposed a stress yield criterion for RC slabs that can consider the shear capacity of concrete and is valid for thin slabs with uniform reinforcement. Based on the yield criterion, Huang Z and Liu XL [15] established a unified model for RC box section members with uniform reinforcement.

In recent years, many studies have focused on the ultimate strength of RC members under combined loading. Huang L and Lu Y [16] studied the overall interactions between different types of loads and established semi-empirical equations for the ultimate strength of symmetrical RC members under combined loading. Rossi and Recupero [17, 18] established analytical formulations for the truss action and arch action, respectively, and calculated the ultimate shear strength of RC members under combined axial forces, bending moments, and shear forces. Panjehpour, Chai, and Voo [19] improved the strut-and-tie model (STM) for deep RC beams using experiments and the finite element method. Different STMs may be established for RC members, and establishing a good STM relies on the experience of calculators. The ultimate strength of RC beams with ratios of shear span to effective depth that are less than 3 can be accurately evaluated using proper STMs.

In this paper, a total of 15 RC beams were fabricated to study the ultimate strength of RC members. These beams had identical geometric parameters and reinforcement. The beams were subjected to different load combinations of axial force, bending moment, shear force and torsion. The experimental results are presented and discussed in detail. This experimental research intends to determine how the ultimate strength of RC members changes with changes in load combinations. Then, a theoretical model was established by determining the shape of the warped failure surface, assuming a proper stress distribution on the failure surface, and considering the equilibrium conditions. The model attempts to develop a concise expression and has a certain level of accuracy for calculating the ultimate strength of RC members subjected to different load combinations. Finally, the accuracy of this model was verified by comparisons with experimental results.

## Experimental design

### RC member design

The length of the 15 experimental beams was 2,200 mm, including a middle experimental segment and two clamped end segments. The experimental segment was 1,200 mm long, and each clamped end was 500 mm long. The cross section of the beams was 240 mm×240 mm. The ratio of the effective length to width *l*_*0*_/*b* was 5, where *l*_*0*_ is the distance between the lateral supports and *b* is the width of the beam. The concrete cover for the outermost reinforcement was 20 mm.

**Fig 1:**
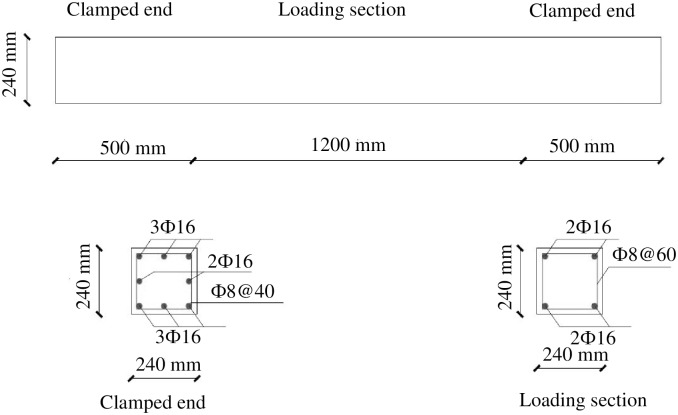
Layout and cross sections of the test beams.

All of the beams were made using the same concrete and reinforcement. Nine concrete test cubes and three samples for each steel species were made to test the material strength. The proportions of concrete mix were 368 kg/m^3^ P.O. 42.5 ordinary Portland cement, 185 kg/m^3^ water, 637 kg/m^3^ medium sand, and 1,184 kg/m^3^ stone. The measured compressive strength of the concrete cubes *f*_*cu*_ was 45.4 N/mm^2^. In the middle experimental segment, the longitudinal steel bars were 4∅16 mm in the corners. The yield strength *f*_*yl*_ and ultimate tensile strength *f*_*ul*_ of the ∅16 mm rebar were 498 and 648 N/mm^2^, respectively. The transverse reinforcements were ∅8@60 mm. The yield strength *f*_*yt*_ and ultimate tensile strength *f*_*ut*_ of the ∅8 mm rebar were 450 and 670 N/mm^2^, respectively. The clamped ends were reinforced with more steel rebar to ensure that the experimental beams failed in the middle experimental segment. The beam dimensions and reinforcement are shown in Fig 1.

### Experimental device

**Fig 2:**
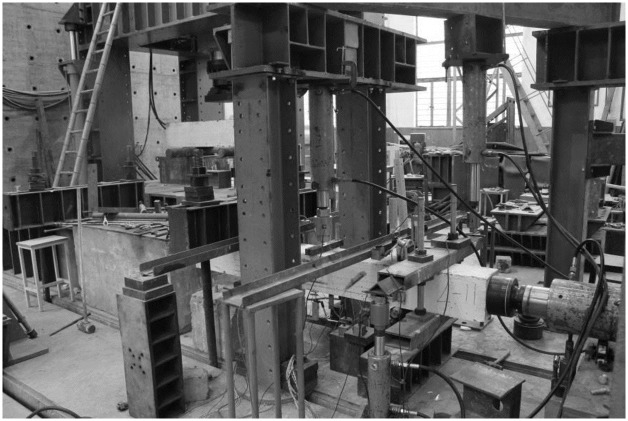
Experimental device.

The experimental device is shown in Fig 2. The beams were supported by two hinged supports at the ends of the middle experimental segment, and the distance between the two supports was 1,200 mm. When torsion was needed, torsion restraints were applied at the left hinged support, and the other hinged support was replaced with a blade bearing to provide vertical support while allowing free rotation. The external forces were applied with 4 hydraulic jacks. A 100 t hydraulic jack was used to apply the axial compressive force. Two 10 t hydraulic jacks and a frame were used to apply torsion. The distance between the two 10 t hydraulic jacks was 1,300 mm. Finally, a 50 t hydraulic jack was used to apply vertical force at the middle segment of the test beams.

### Loading procedure

**Fig 3:**
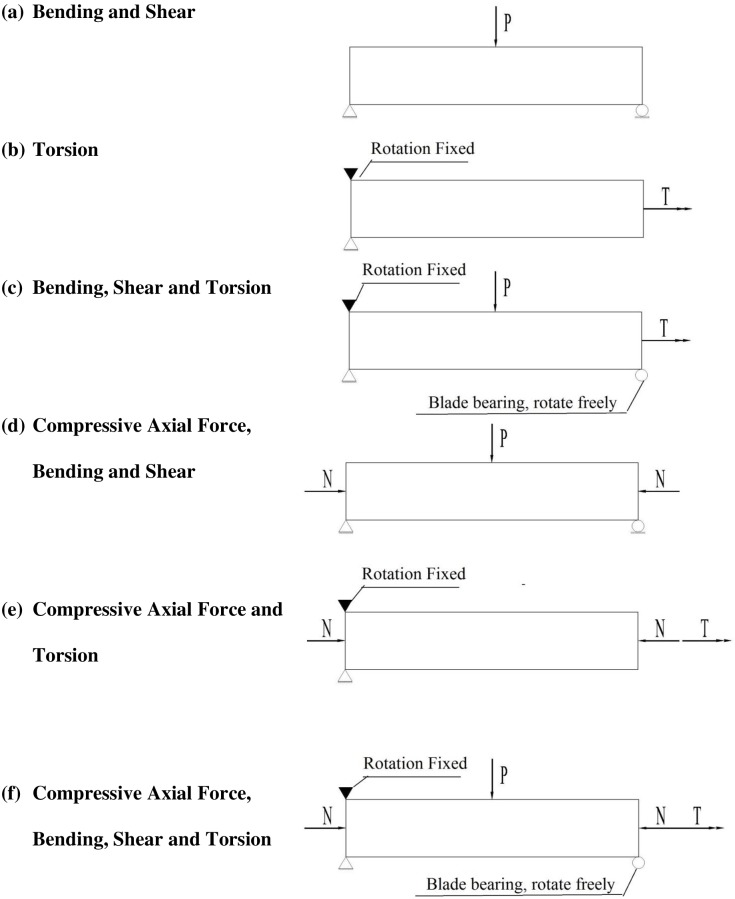
Combinations of loads and supports.

The 15 experimental beams were divided into four groups based on the different load combinations of axial force (*N*), bending (*M*), shear (*V*) and torsion (*T*). The first two groups had load combinations without axial force (*N*), and the last two groups had load combinations with axial force (*N*). The first group consisted of 2 beams under bending and shear (MV×2) and 2 beams under pure torsion (T×2). The second group consisted of 4 beams under bending, shear and torsion (MVT×4). The third group consisted of 1 beam under axial force, bending and shear (NMV×1) and 1 beam under axial force and torsion (NT×1). The last group consisted of 5 beams under axial force, bending, shear and torsion (N_0.15_MVT×2 and N_0.3_MVT×3). The shear span ratio was 3 for all of the load combinations. All of the load combinations and supports are shown in Fig 3.

The loads were slowly applied incrementally. The loading sequence was compressive axial force, torsion and vertical force. The load increment of each load step was 60 kN for compressive axial force and 1.3 kN•m for torsion. When applying vertical force to the beam, the load increment of each load step was 5 kN before the concrete cracked, 10 kN after cracking occurred and 5 kN when the loads approached 80% of the estimated ultimate strength of the RC beam. Each load step was maintained for 5 min, and the concrete strain, middle deformation and crack developments were observed and recorded at each load step.

## Experimental results

**Fig 4:**
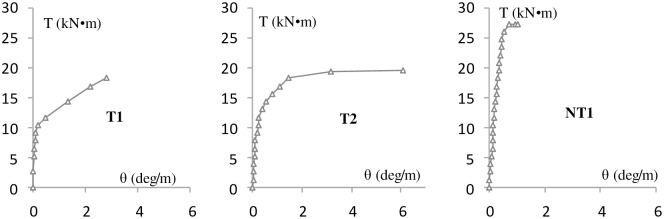
T-θ curves of the experimental beams.

**Fig 5:**
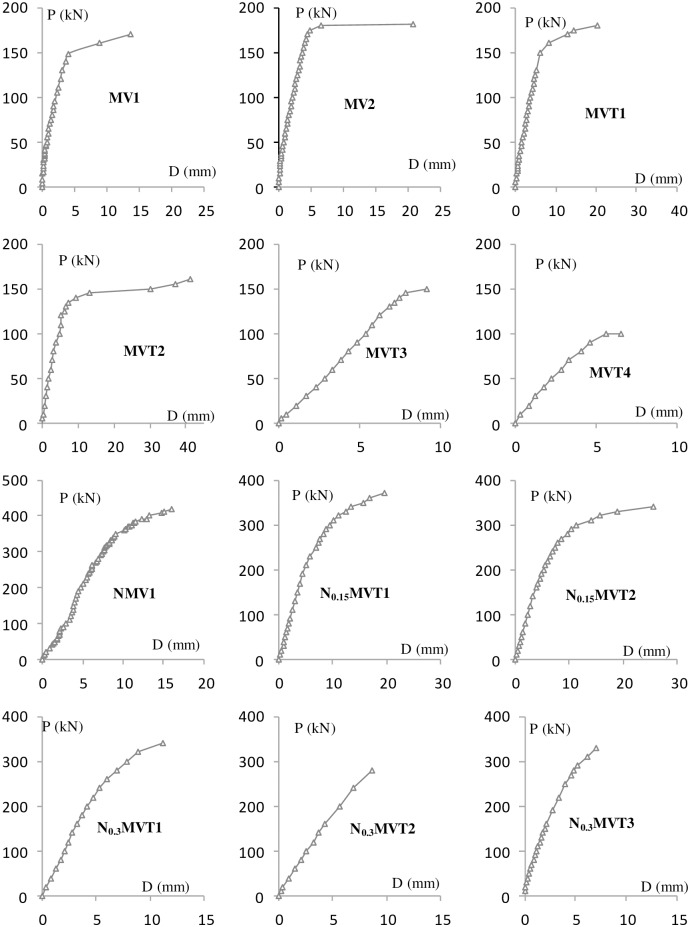
P-Δ curves at the mid-spans.

**Fig 6:**
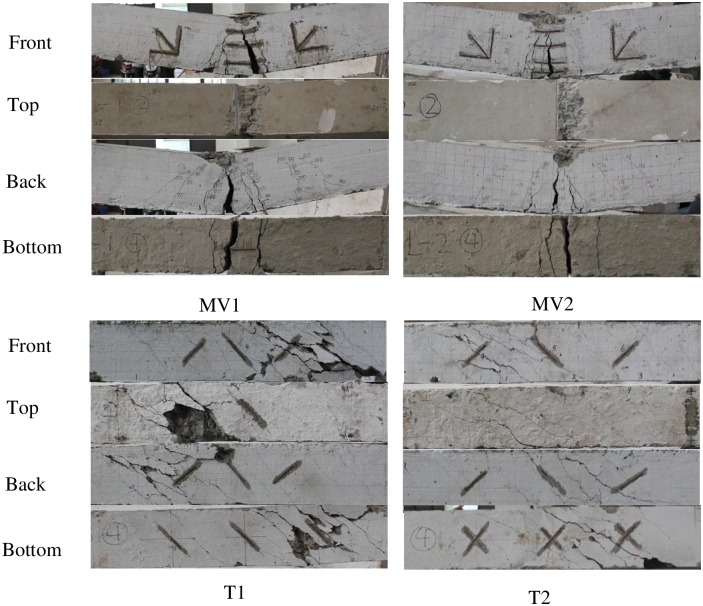
Crack graphs of Group 1.

**Fig 7:**
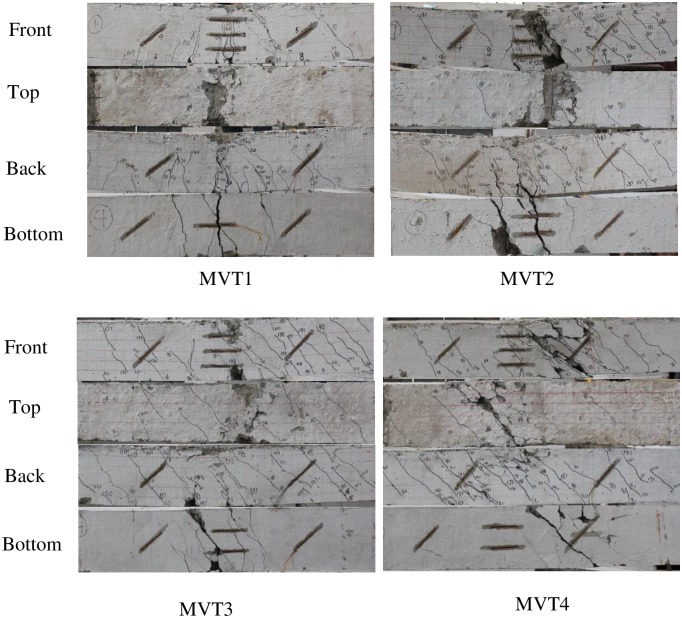
Crack graphs of Group 2.

**Fig 8:**
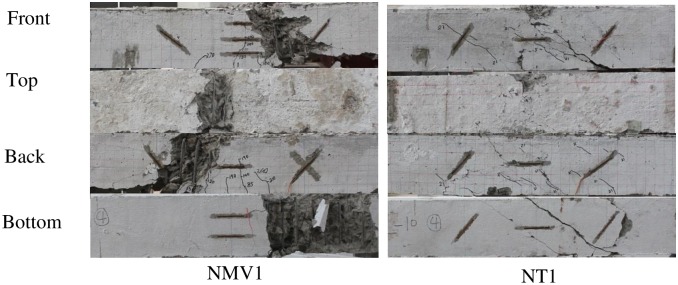
Crack graphs of Group 3.

**Fig 9:**
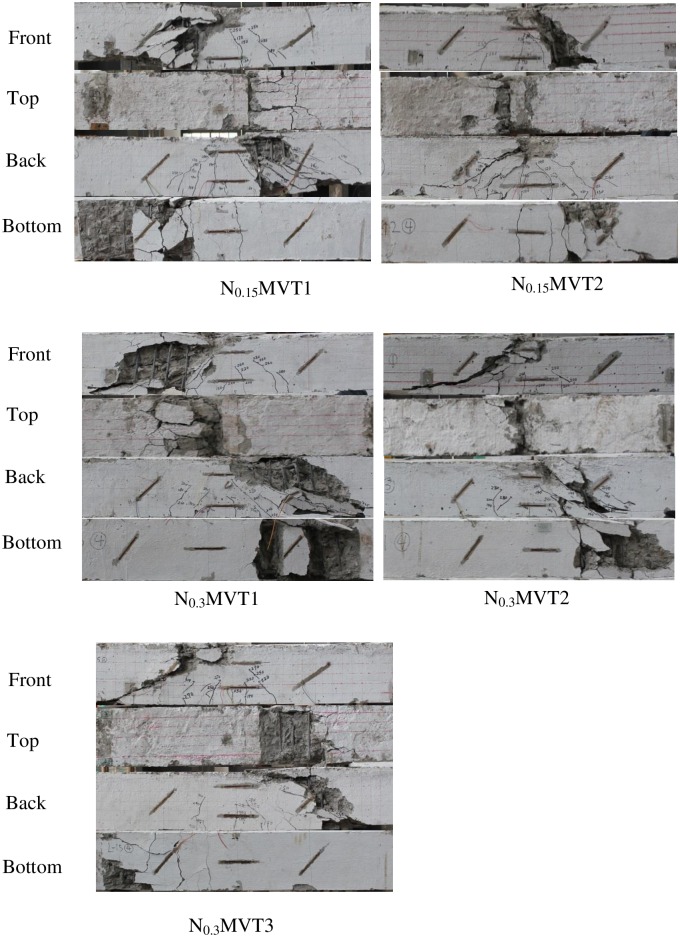
Crack graphs of Group 4.

The experimental results are shown in Figs 4–9 and Table 1. Fig 4 shows the T-θ curves of the experimental beams. Fig 5 shows the P-Δ curves at the mid-spans. Figs 6–9 show the experimental crack graphs. Table 1 lists the cracking loads and the ultimate loads of the experimental beams.

**Table 1:** Summary of the experimental results.

Specimen Name	Cracking loads				Ultimate loads			
	N_cr_ (kN)	M_cr_ (kN⋅m)	V_cr_ (kN)	T_cr_ (kN⋅m)	N_u_ (kN)	M_u_ (kN⋅m)	V_u_ (kN)	T_u_ (kN⋅m)
MV1	-	12.9	21.5	-	-	52.5	87.5	-
MV2	-	11.4	19.0	-	-	54	90	-
T1	-	-	-	10.40	-	-	-	18.46
T2	-	-	-	5.20	-	-	-	19.76
MVT1	-	7.2	12	6.69	-	55.2	92	6.69
MVT2	-	0	0	6.50	-	48	80	10.51
MVT3	-	0	0	9.10	-	45	75	14.33
MVT4	-	0	0	7.80	-	33	55	14.33
NMV1	-588	25.5	42.5	-	-588	129	215	-
NT1	-588	-	-	13.65	-588	-	-	28.60
N_0.15_MVT1	-294	27	45	10.51	-294	90	150	10.51
N_0.15_MVT2	-294	36	60	14.33	-294	84	140	14.33
N_0.3_MVT1	-588	48	80	10.51	-588	102	170	10.51
N_0.3_MVT2	-588	42	70	14.33	-588	90	150	14.33
N_0.3_3MVT3	-588	51	85	14.33	-588	99	165	14.33

### Group 1 (MV and T)

Group 1 consists of four beams, which are referred to as MV1, MV2, T1 and T2. Beam MV1 was subjected to bending moment and shear force and was supported by two hinged supports. A 50 t hydraulic jack was used to apply a vertical force at the middle of the beam. In the initial loading procedure, the strain gages indicated that the concrete strain increased linearly in the beam’s middle segment. The first crack appeared on the bottom when the shear force *V* reached 21.5 kN. The concrete on the bottom stopped carrying load because of the cracks. Along with the loading, new inclined cracks gradually appeared between the supports and the loading point due to the influence of shear stress. No additional cracks were observed when the internal force was rearranged. The existing cracks grew longer and wider until one crack became the critical crack. The longitudinal reinforcement across the critical crack yielded, and MV1 failed when the shear force reached 87 kN. The failure procedure of beam MV2 was similar to that of MV1.

Beams T1 and T2 were loaded under pure torsion. The left end of the beam was fixed, and the other end was supported by a blade bearing to supply the torsional load. The distance between the two supports was maintained at 1,200 mm. Two 10 t hydraulic jacks and a frame were used to apply torsion. The value of the force arm was 1,300 mm for the two jacks. In the initial loading procedure, the strain gages and the load-rotation curve indicated that the concrete worked in the elastic stage. Then, the first crack was observed when the torsion reached 10.4 kN•m for T1 and 5.2 kN•m for T2. Along with the torsional increase, additional cracks with inclination angles of 45° gradually appeared and developed to form spiral cracks. The cracked concrete carried less loads, and the reinforcement across the cracks gradually began to yield. After the reinforcement yielded, the beam rotation increased more rapidly. Finally, the front concrete of T1 and the bottom concrete of T2 were crushed.

### Group 2 (MVT)

In Group 2, four beams, MVT1, MVT2, MVT3 and MVT4, were loaded under a combination of bending moment, shear force and torsion. The supports were the same as those of beams T1 and T2. First, the torsion was first applied by two 10 t hydraulic jacks. Then, the bending moment and shear force were applied by one 50 t hydraulic jack applying a vertical force P at the middle of the beams. The torsion applied to MVT1, MVT2, MVT3 and MVT4 was 0.35*T*_*u*_, 0.55*T*_*u*_, 0.75*T*_*u*_ and 0.75*T*_*u*_, respectively. The value of *T*_*u*_ was set to the mean value of the ultimate torsion of beams T1 and T2 found from the Group 1 results.

The experiment results indicated that the ultimate strength of bending and shearing decreased with increasing applied torsion force.

MVT1 did not crack under torsion of 0.35*T*_*u*_. The ultimate strength of MVT1 was very similar to those of MV1 and MV2, and the failure modes were similar. Therefore, the torsion of 0.35*T*_*u*_ was sufficiently small that the applied torsion did not significantly influence the ultimate strength of the beam.

MVT2 cracked when the torsion reached 6.5 kN⋅m. With the the torsion and vertical loading, inclined cracks were observed between the loading point and supports. These cracks mainly resulted from the shear stress in the concrete, and the inclination angles were approximately 45°. Cracks were observed in the lower part of the beam near the middle segment. These cracks mainly resulted from the bending moment, and the inclination angles were approximately 60–70°. From the load-deformation curve of the mid-span, as shown in Fig 5, the deformation started to increase rapidly when the shear force reached 67.5 kN, indicating the yielding of the longitudinal reinforcement. When the shear force reached 80 kN, the concrete in compression was crushed, and MVT2 reached its ultimate strength.

MVT3 and MVT4 cracked when the torsion reached 9.1 and 7.8 kN, respectively. When the torsion increased to 0.75*T*_*u*_, cracks gradually developed and appeared as spiral cracks. With further increases in the vertical force, the concrete was crushed in compression.

### Group 3 (NMV and NT)

In Group 3, Beam NMV1 was tested under combined axial force, bending moment and shear force. The supports of NMV1 were the same as those for MV1 and MV2. A 50 t hydraulic jack was used to apply the vertical force, just as in the experiments for MV1 and MV2. A 100 t hydraulic jack was used to apply the axial compressive force on the NMV1 beam. In the first loading procedure, the axial force was gradually increased to 588 kN, which was equal to 30% of the total axial bearing capacity (*0*.*3f*_*c*_*A*). Next, the vertical force was loaded to apply the bending moment *M* and shear force *V* to NMV1. The first crack was observed when the shear force *V* reached 42.5 kN. The cracking load of NMV1 was greater than those of MV1 and MV2. As the vertical force increased, more cracks appeared between the middle loading point and the supports until an inclined crack finally became the critical crack. The inclination angle of the critical crack was approximately 28° on the front. The concrete in the compressive area was crushed when the shear force reached 215 kN.

Beam NT1 was loaded under combined axial force and torsion. The supports were the same as those of T1 and T2. One 100 t hydraulic jack was added to apply an axial compressive force. Two 10 t hydraulic jacks were used to apply torsion to the beam in the same manner as in the experiments for T1 and T2. First, an axial compressive force of 588 kN was applied. Then, a torsion load was applied and increased.

The first crack of beam NT1 was observed considerably later than those of T1 and T2. After the concrete cracked, the internal stress was transferred to the reinforcement. The torsion-rotation curve illustrates that the rotation of NT1 began increasing rapidly when the torsion reached 26 kN•m, indicating the yielding of the reinforcement. The critical inclined crack of NT1 appeared on the bottom, and the inclination angle of the crack was approximately 35°. The ultimate torsion of NT was 28.6 kN•m.

### Group 4 (NMVT)

Group 4 consisted of five beams, N_0.15_MVT1, N_0.15_MVT2, N_0.3_MVT1, N_0.3_MVT2 and N_0.3_MVT3, which were loaded under axial forces, bending moments, shear forces and torsion. The supports were the same as those for beams MVT. The axial force was applied first, followed by torsion and then vertical force.

The axial compressive force on N_0.15_MVT1 and N_0.15_MVT2 was 294 kN, which was equal to 0.15*f*_*c*_*A*. The torsion on N_0.15_MVT1 was 0.55*T*_*u*_, and the torsion on N_0.15_MVT2 was 0.75*T*_*u*_. The failure procedures of N_0.15_MVT1 and N_0.15_MVT2 were similar, but the ultimate strength of N_0.15_MVT1 was higher. No cracks were observed when only axial force and torsion were applied. The compressive force caused N_0.15_MVT1 and N_0.15_MVT2 to crack later than MVT2, MVT3 and MVT4. The cracks were flexural and shear cracks and developed more severely in the additive shear stress zone with *τ*_*v*_*+τ*_*T*_.

The inclination angles of the cracks were smaller than those of MVT2, MVT3 and MVT4 due to the existence of normal axial compressive stress. The concrete covers started to peel off due to these cracks in the additive shear stress zone with *τ*_*v*_*+τ*_*T*_. The load-deformation curves of the middle segment indicate that N_0.15_MVT1 and N_0.15_MVT2 still showed notable ductility behaviour during loading.

The axial compressive forces on N_0.3_MVT1, N_0.3_MVT2 and N_0.3_MVT3 were 588 kN, which was equal to 0.3*f*_*c*_*A*. The torsion on N_0.3_MVT1 was 0.55*T*_*u*_. The torsion on N_0.3_MVT2 and N_0.3_MVT3 was 0.75*T*_*u*_. The cracking modes of N_0.3_MVT1, N_0.3_MVT2 and N_0.3_MVT3 were similar to those of N_0.15_MVT1 and N_0.15_MVT2, but the higher compressive force made the inclination angles of cracks even lower in N_0.3_MVT1, N_0.3_MVT2 and N_0.3_MVT3. Furthermore, the higher compressive force caused N_0.3_MVT1, N_0.3_MVT2 and N_0.3_MVT3 to exhibit brittle behaviour during the loading and failure.

### Discussion of the results

The ultimate strength of RC members is affected by many factors, such as the material properties, reinforcement ratio, member sizes, loading methods, shear span ratio and loading combination. This paper focuses on the influence of different loading combinations on the ultimate strength. The result is appropriate for members with ordinary material, normal reinforcement, and normal span ratios.

The following conclusions were drawn from the research and experimental results:

1. Torsion forces could reduce the ultimate capacity of beams under combined loading due to three main factors. First, torsion can increase the tension stress of the longitudinal reinforcement and cause a compression zone on the opposite side. Second, shear stress caused by torsion can decrease the concrete compressive strength. Finally, torsion may peel off the concrete covers under high shear stress.
2. The compressive axial force could affect the ultimate strength of the beams. An appropriate compressive force can increase the ultimate strength of the beams by decreasing the tensile stress of the longitudinal reinforcement and could work as additional longitudinal reinforcement in the tension zone. Furthermore, an appropriate compressive force could improve the shear strength of the concrete. However, a high axial compressive force may decrease the ultimate strength of the beams by increasing the compressive stress in the concrete compression zone and may crush the compressive concrete before failure of the tensile longitudinal reinforcement.
3. Cracking angles change with changes in the load combinations. The critical cracks constitute the twist failure surface of the beams, and the twist failure surface affects the ultimate strength of the beams. When the bending moment dominates the combined load, the inclination angles of the critical cracks are approximately 90°, such as in beams MV1 and MV2. When pure torsion or shear force dominates the combined load, the inclination angles of the critical cracks are approximately 45°. When an axial compressive force is applied, the axial force changes the direction of the principal stress in the concrete and thus the cracking angles.

## Theoretical approach to the failure model

Based on the above experimental research on 15 RC beams under combined axial force, bending, shear and torsion loading, a simplified failure model is established as the theoretical approach. This failure model corresponds well with the above experimental research results and is fairly accurate. Additionally, the model attempts to provide a concise expression for engineering applications.

### Basic assumptions

The ultimate strength of RC members under combined loading is highly complex. The following basic assumptions are adopted to simplify the calculation of the failure model.

1. The effect of the load path was neglected. The model is suitable for members whose ultimate strengths are not highly sensitive to the load path.
2. The dowel action of the rebar was neglected.
3. The stresses of the longitudinal reinforcement were calculated based on the Bernoulli truss model. There are two classic models for the shear strength of RC beams, namely, the STM and the Bernoulli truss model. The STM is more appropriate for expressing the force transfer mechanism when the shear span ratio is λ<3. The stress distribution is even throughout the majority of the beam when the shear span ratio is λ≥3; thus, the Bernoulli truss model is more appropriate. In most engineering applications, the shear span ratios of the majority of beams and columns are no less than 3. Thus, the Bernoulli truss model was adopted in the model.
4. The stresses of the transverse web reinforcement across the failure surface are assumed to reach the yield strength. When the bending moment dominated the combined load, the transverse web reinforcement may not yield. In this situation, a reduction factor ∅ is introduced to approximately consider the real stress in the web reinforcement.

### Formulations of the proposed model

**Fig 10:**
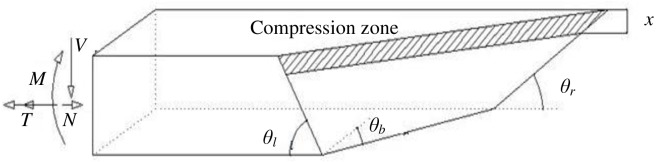
Warped failure surface.

The simplified failure model is based on the ultimate equilibrium method. The first step for establishing the model is to determine the warped failure surface. As shown in Fig 10, 4 parameters were adopted to describe the surface: the three critical crack angles *θ*_*l*_, *θ*_*r*_, and *θ*_*b*_ and the depth of the compressive concrete zone *x*. In reality, the depth of the compressive zone is different on the left and right sides, but a mean depth of *x* is used to simplify the expression.

The depth of the compression zone *x* can be calculated based on the Bernoulli truss model as follows:

$$N=\sigma_{s}A_{s}-f_{y}^{\prime }A_{s}^{\prime }-f_{c}bx$$ (1)

$$\sigma_{s}=E_{s}\varepsilon_{cu}(\frac{h_{0}}{x}-1)$$ (2)

where *A*_*s*_ is the area of longitudinal reinforcement in the tension area, *A*_*s*_’ is the area of longitudinal reinforcement in the compression area, *b* is the width of the beam section, *x* is the depth of the compression zone, *σ*_*s*_ is the stress in the tensile reinforcement, *f*_*y*_*’* is the yield strength of the compressive reinforcement, *f*_*c*_ is the compressive strength of concrete, *E*_*s*_ is the modulus of elasticity of the reinforcement, *ε*_*cu*_ is the ultimate compressive strain of concrete, and *h*_*0*_ is the effective height of the section, which is the distance from centre of tensile reinforcement to the opposite edge.

**Fig 11:**
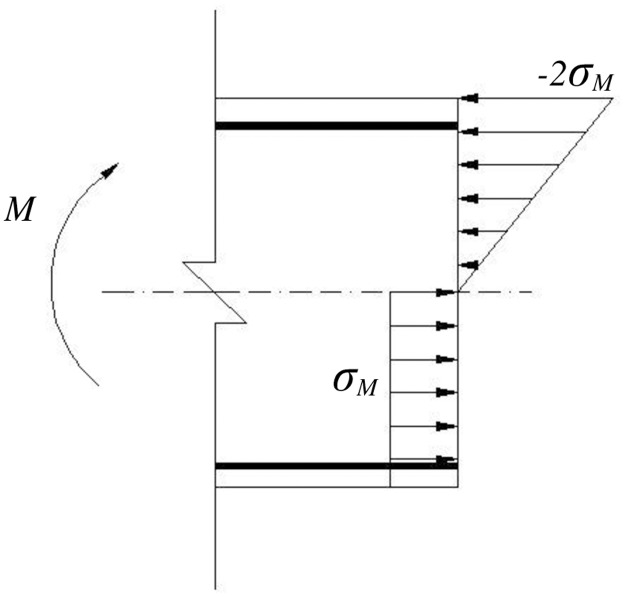
Stress distributions on the section under bending.

The stress distribution in the section under bending is assumed to be as shown in Fig 11. The stress on the section under torsion is assumed to be distributed along the external box section. The width of the box section *t* is calculated based on the results of Rahal and Collins’ model [20]. The stress under different single external forces can be expressed as follows:

1. Axial force
  $$\sigma_{N}=\frac{N}{bh}$$ (3)
2. Bending moment
  $$\sigma_{M}=\frac{24M}{7bh^{2}}$$ (4)
3. Shear force
  $$\tau_{V}=\frac{V}{bh}$$ (5)
4. Torsion
  $$\tau_{T}=\frac{T}{2A_{cor}t}$$ (6)
  where *t* is calculated based on Rahal and Collins’s model.
  $$\begin{array}{ll} t & =\frac{0.833bh}{4(b+h)}\\ A_{cor} & =b_{cor}\times h_{cor}\\ b_{cor} & =b-t\\ h_{cor} & =h-t \end{array}$$

The tensile stress caused by the bending moments is mainly undertaken by the longitudinal reinforcement. Thus, the tensile stresses in the left and right flanges can be regarded as the stresses from the axial force. In each flange, the stresses can be expressed as follows:

$$\begin{array}{l} \sigma_{l}=\frac{N}{bh},\tau_{l}=\frac{T}{2A_{cor}t}+\frac{V}{bh}\\ \sigma_{r}=\frac{N}{bh}\;,\tau_{r}=\frac{T}{2A_{cor}t}-\frac{V}{bh}\\ \sigma_{b}=\frac{N}{bh}+\frac{24M}{7bh^{2}},\tau_{b}=\frac{T}{2A_{cor}t} \end{array}$$ (7)

The model uses the directions of the first cracks as the directions of the warped failure surface. The cotangents of the crack inclination angles can be calculated using Eq 8.

$$\text{cot}\theta_{i}=\frac{2\tau_{i}}{\sqrt{\sigma_{i}^{2}+4\tau_{i}^{2}}+\sigma_{i}},i=l,r,b$$ (8)

The second step to establish the model is to determine the stresses of the longitudinal and transverse reinforcement across the failure surface. The stresses of the longitudinal reinforcement can be obtained from Eq 2. If shear or torsion dominates the combined load, then the transverse reinforcement is assumed to yield when the member reaches its ultimate strength. However, the transverse reinforcement may not yield if the reinforcement is excessive or if the shear force and torsion are small. Thus, a factor ∅ is used to reduce the stress in the model. To simplify the model, the factor ∅ is calculated using Eq 9. This equation is based on the ratio of the external forces to the ultimate forces of the transverse reinforcement across the failure surface in the left flange. Because the stress of the transverse reinforcement in the left flange is no less than that in the right flange, this simplification tends to be conservative.

$$\phi =\frac{\left|\frac{T-T_{c}}{2A_{0}}h_{cor}+\frac{V-V_{c}}{2}\right|}{n_{l}f_{yv}A_{sv1}}\leq 1$$ (9)

where *n*_*l*_ is the number of reinforcements across the crack in the left flange, *T*_*c*_ is the resistant torsion of concrete and *V*_*c*_ is the resistant shear force of concrete. The calculations of *n*_*l*_, *T*_*c*_ and *V*_*c*_ is given in equations later in this chapter.

The third step is to establish the formulation through the ultimate equilibrium. Eq 10 can be obtained from the equilibrium of the failure surface in the left zone.

$$\begin{array}{l} \frac{T}{2A_{cor}}h_{cor}+\frac{V}{2}=n_{l}\phi A_{sv1}f_{yv}+F_{l} \end{array}$$ (10)

where

$$\begin{array}{l} F_{l}=\frac{bh_{0}}{2}\tau_{c}+h_{cor}t\tau_{c}\\ n_{i}=\frac{h_{cor}cot\theta_{i}}{s},i=l,r,b \end{array}$$

In Eq 10, the shear strength of concrete *τ*_*c*_ is calculated based on the Tasuji-Slate-Nilson. failure criterion. The factor γ is added to consider the strength reduction after cracking. According to experimental research, γ is set to 0.5.

$$\tau_{c}=\;\frac{\;\gamma \sqrt{f_{c}^{2}+(1-f_{c}/f_{t})f_{c}\sigma -f_{c}/f_{t}\sigma^{2}}}{1+(f_{c}/f_{t})}\;$$ (11)

Similarly,

$$\begin{array}{l} \frac{T}{2A_{cor}}h_{cor}-\frac{V}{2}=n_{r}\phi A_{sv1}f_{yv}+F_{r}\\ \frac{T}{2A_{cor}}b_{cor}=n_{b}\phi A_{sv1}f_{yv}+F_{b} \end{array}$$ (12)

where

$$\begin{array}{l} F_{r}=-\frac{bh_{0}}{2}\tau_{c}+h_{cor}t\tau_{c}\\ F_{b}=b_{cor}t\tau_{c} \end{array}$$

Eq 13 can be established from the bending moment equilibrium at the centre of the compression zone.

$$\begin{array}{l} M+N\left(\frac{h}{2}-\frac{x}{2}\right)=\sigma_{s}A_{s}\left(h_{0}-\frac{x}{2}\right)+f_{y}^{\prime }A_{s}^{\prime }\left(\frac{x}{2}-a_{s}^{\prime }\right)-n_{l}A_{sv1}\phi f_{yv}(\frac{h_{cor}\text{cot}\theta_{l}}{2}+\frac{b_{cor}\text{cot}\theta_{b}}{2})\\ -n_{r}A_{sv1}\phi f_{yv}(\frac{h_{cor}\text{cot}\theta_{r}}{2}+\frac{b_{cor}\text{cot}\theta_{b}}{2}) \end{array}$$ (13)

By substituting Eqs 10–12 into Eqs 13 and 14 can be expressed as a unified expression:

$$\begin{array}{l} \frac{N}{N_{0}}+\frac{M}{M_{0}}+\frac{{\left(V-V_{c}\right)}^{2}}{V_{0}^{2}}+\frac{{\left(T-T_{c}\right)}^{2}}{T_{0}^{2}}=1 \end{array}$$ (14)

where

$$\begin{array}{ll} N_{0} & =\left[\sigma_{s}A_{s}\left(h_{0}-\frac{x}{2}\right)+f_{y}^{\prime }A_{s}^{\prime }\left(\frac{x}{2}-a_{s}^{\prime }\right)\right]/\left(\frac{h}{2}-\frac{x}{2}\right)\\ M_{0} & =\sigma_{s}A_{s}\left(h_{0}-\frac{x}{2}\right)+f_{y}^{\prime }A_{s}^{\prime }\left(\frac{x}{2}-a_{s}^{\prime }\right)\\ V_{0}^{2} & =4\phi A_{sv1}f_{yv}\left[\sigma_{s}A_{s}\left(h_{0}-\frac{x}{2}\right)+f_{y}^{\prime }A_{s}^{\prime }\left(\frac{x}{2}-a_{s}^{\prime }\right)\right]/s\\ T_{0}^{2} & =4\phi A_{sv1}f_{yv}A_{cor}^{2}\left[\sigma_{s}A_{s}\left(h_{0}-\frac{x}{2}\right)+f_{y}^{\prime }A_{s}^{\prime }\left(\frac{x}{2}-a_{s}^{\prime }\right)\right]/\left[sh_{cor}(b_{cor}+h_{cor})\right]\\ V_{c} & =bh_{0}\tau_{c}\\ T_{c} & =2A_{cor}t\tau_{c} \end{array}$$

where *a*_*s*_^*’*^ is the distance from the centre of the compressive reinforcement to the nearby edge.

The proposed model is different from that given in existing studies. Table 2 shows the differences between the proposed model and models from the literature.

**Table 2:** Differences between the proposed model and models from the literature.

Model	Theory	Section	Load Combination	Method
DSFM [5–7]	Truss theory	Slab; Box section	All load combinations	Solve simultaneous equations of equilibrium condition, deformation compatibility condition and constitutive relationship
Soften truss model [8–10]	Truss theory	Slab; Box section	All load combinations	Solve simultaneous equations of equilibrium condition, deformation compatibility condition and constitutive relationship
Huang L’s model [16]	Empirical equations	Rectangular section	All load combinations	Statistical analysis method
Rossi and Recupero's models [17–18]	Limit analysis	Rectangular section; Circular section	Axial force, bending moment, and shear force	Establish analytical formulations for the truss action and arch action and then calculate the ultimate shear strength of RC members
Panjehpour, Chai, and Voo’s model [19]	STM	Rectangular section	Bending moment and shear force	Establish an STM for members based on load-transferring mechanism
Huang Z’s model [12, 15]	Limit analysis	Slab; Box section	All load combinations	Establish yield equations based on the stress yield criterion [14] for RC slabs
Model proposed in this work	Limit analysis	Rectangular section	All load combinations	Determine the shape of the warped failure surface, assume the stress distribution on the failure surface, and then establish equations based on the equilibrium condition

## Comparisons between the proposed theoretical model and experimental results

Table 3 shows the experimental results and the calculation results based on the proposed theoretical model. The bending moment at the intersection point of the first inclined crack and the longitudinal reinforcement were used in the calculation. This intersection point was approximately *h*_*0*_ from the middle span of the beam. The concrete covers may peel off when the members are under pure torsion or 0.75*T*_*u*_ of pure torsion. Thus, the value of *A*_*cor*_ should be decreased; 0.6*A*_*cor*_ was used in the calculation.

**Table 3:** Comparison of the experimental results and theoretical model results.

Specimen Name	N_exp_ (kN)	M_exp_ (kN⋅m)	V_exp_ (kN⋅m)	T_exp_ (kN⋅m)	N_calc_ (kN)	M_calc_ (kN⋅m)	V_calc_ (kN⋅m)	T_calc_ (kN⋅m)	V_calc_/V_exp_ T_calc_/T_exp_
MV1	-	52.5	87.5	-	-	51.38	85.64	-	0.98
MV2	-	54	90	-	-	51.38	85.64	-	0.95
T1	-	-	-	18.46	-	-	-	18.97	1.03
T2	-	-	-	19.76	-	-	-	18.97	0.96
MVT1	-	55.2	92	6.69	-	51.03	85.05	6.69	0.92
MVT2	-	48	80	10.51	-	49.40	82.34	10.51	1.03
MVT3	-	45	75	14.33	-	35.27	58.78	14.33	0.78
MVT4	-	33	55	14.33	-	35.27	58.78	14.33	1.07
NMV1	-588	129	215	-	-588	113.26	188.77	-	0.88
NT1	-588	-	-	28.60	-588	-	-	30.07	1.05
N_0.15_MVT1	-294	90	150	10.51	-294	85.67	142.79	10.51	0.95
N_0.15_MVT2	-294	84	140	14.33	-294	78.08	130.13	14.33	0.93
N_0.3_MVT1	-588	102	170	10.51	-588	109.79	182.98	10.51	1.08
N_0.3_MVT2	-588	90	150	14.33	-588	98.99	164.98	14.33	1.10
N_0.3_MVT3	-588	99	165	14.33	-588	98.99	164.98	14.33	1.00
Average									0.98
Standard deviation									0.08
Variation coefficients									8.54%

**Fig 12:**
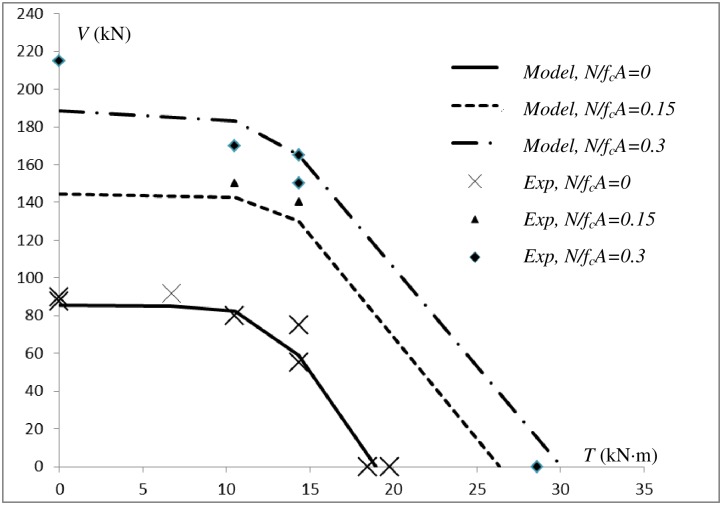
Comparison of the proposed model results and experimental results.

Fig 12 compares the comparison between the proposed theoretical model results and the experimental results. This figure illustrates that the model coincides well with the experimental results for the majority of members. Only NMV1 and MVT3 deviate noticeably from the experimental results. For NMV1, the model result is 12% lower than the experimental result. This difference may be caused by the hardening of the longitudinal reinforcement. Considering the discreteness of the concrete experimental results, this error is acceptable. MVT3 and MVT4 experience the same conditions. For MVT4, the calculated result coincides well with the experiment result. For MVT3, the calculated result is 22% lower than the experimental result. Fig 7 illustrates that the external concrete of MVT4 was damaged more seriously than that of MVT3, as the external concrete of MVT3 was still solid. If *A*_*cor*_ was used in the calculation instead of 0.6*A*_*cor*_, then calculated result for MVT3 is 78.34 kN, which is closer to the experimental result of 75 kN. This result indicates that the external concrete may occasionally peel off when approximately 75% of the pure torsion strength is applied and that the experimental results show discreteness. The lower calculation value should be used for safety.

## Conclusions

This paper conducts experimental research on the ultimate strength of RC members. Fifteen beams with identical rectangular sections and reinforcement were tested. These beams were divided into 4 groups and subjected to different combinations of axial force, bending moment, shear force and torsion. The ultimate strength, load-deformation behaviour and cracking graphs were recorded, and the experimental results were discussed. Then, a unified theoretical model for estimating the strength of these beams based on the limit equilibrium analysis was proposed.

The following conclusions can be drawn from this study:

1. The experimental research demonstrates how the ultimate strength of RC members changes due to changes in combined loads. The experimental results conform to common sense and can serve as a benchmark for theoretical analysis.
2. Torsion can reduce the ultimate capacity of beams under combined loading, and the axial compressive force could affect the ultimate strength of beams. An appropriate compressive force can increase the ultimate strength of the beam and the shear strength of the concrete. However, a high compressive axial force may decrease the ultimate strength of the beam.
3. The crack inclination angles change with changes in combined load. The critical cracks constitute the twist failure surface of the beams, which affects the ultimate strength of the beams. When a compressive axial force is applied, the axial force changes the direction of the principal stress in the concrete and thus changes the crack inclination angles.
4. According to the experimental research, a unified theoretical model was established by determining the shape of the warped failure surface, assuming an appropriate stress distribution on the failure surface, and considering the equilibrium conditions. The model focuses on the influence of axial force on the spatial angle of the ultimate failure surface of members, on the brittle or ductile failure model, and on the ultimate capacity of the members. Based on the limit equilibrium analysis, the geometric shape and detailed dimensions of the failure surface of an RC member can be determined for varying combined loads.
5. The unified theoretical formulas can be simplified into commonly accepted formulas for RC members subjected to only one force of the four loads: a single axial force, bending, shear or torsion. The formulas can also be used to calculate the combined ultimate strength under combined loading, which illustrates that the unified theoretical model is a more general model and confirms the validity of the model.
6. The accuracy of the proposed unified theoretical model is proven with experimental research. The agreement between the experimental results and model illustrates that the proposed theoretical model can accurately estimating the ultimate strength of rectangular RC members under combined axial force, bending, shear and torsion loading.
